# Monitoring translation in synaptic fractions using a ribosome profiling strategy

**DOI:** 10.1016/j.jneumeth.2019.108456

**Published:** 2020-01-01

**Authors:** Konstanze Simbriger, Inês S. Amorim, Kleanthi Chalkiadaki, Gilliard Lach, Seyed Mehdi Jafarnejad, Arkady Khoutorsky, Christos G. Gkogkas

**Affiliations:** a Centre for Discovery Brain Sciences, University of Edinburgh, EH8 9XD, Edinburgh, Scotland, UK; b Patrick Wild Centre, EH8 9XD, Edinburgh, Scotland, UK; c Centre for Cancer Research and Cell Biology, The Queen’s University of Belfast, BT9 7AE, Belfast, Northern Ireland, UK; d Department of Anesthesia, Faculty of Dentistry and Alan Edwards Centre for Research on Pain, McGill University, H3A 0G1, Montréal, QC, Canada; e Simons Initiative for the Developing Brain, EH8 9XD, Edinburgh, Scotland, UK

**Keywords:** GO, gene ontology, RPF, ribosome-protected fragment, TE, translational efficiency, TRAP, translating ribosome affinity purification, SN, synaptoneurosomes, Synapse, Synaptoneurosomes, mRNA translation, Ribosome profiling, Local translation

## Abstract

**Background:**

The aim of this study was to develop a method to study genome-wide local translation in biochemically isolated synaptic fractions (synaptoneurosomes). This methodology is of particular interest for neurons, due to the cardinal role of local translational control in neuronal sub-compartments, such as dendrites, for plasticity, learning, memory, and for disorders of the nervous system.

**New method:**

We combined established methods for purifying synaptoneurosomes with translational profiling (ribosome profiling), a method that employs unbiased next generation sequencing to simultaneously assess transcription and translation in a single sample.

**Results:**

The two existing methods are compatible to use in combination and yield high quality sequencing data, which are specific to synaptic compartments. This new protocol provides an easy to implement workflow, which combines biochemical isolation of synaptoneurosomes of varying levels of purity (crude or Percoll gradient purified) with the use of a commercial kit to generate sequencing libraries.

**Comparison with existing methods:**

Compared to previous studies of the synaptic translatome, our method shows less contamination with non-neuronal cell types or non-synaptic compartments, increasing the specificity of the data obtained.

**Conclusions:**

Combining the isolation of functional synaptic units with ribosome profiling offers a powerful tool to study local translation in synaptic compartments both in health and disease.

## Highlights

• Ribosome profiling in synaptosomes.
• Transcriptome and translatome profiling from synaptic fractions.
• Powerful tool to study local translation at the synapse.

## 1 Introduction

Neurons are highly specialised cells, which require tight spatial and temporal control of protein synthesis, in order to maintain their functionality and efficiently respond to neuronal activity and external stimuli. Since neuronal compartments, such as dendrites and pre-synaptic terminals, are often situated at great distances from the cell soma, neurons have developed mechanisms to transport mRNAs in a suppressed state to different remote compartments within the cell and allow initiation of protein synthesis in a local and timely manner (Rangaraju et al., 2017). Local translation is important for a variety of cellular mechanisms, including axonal guidance, growth cone development and synaptic plasticity, thus influencing neuronal development and crucial processes such as learning and memory (Holt and Schuman, 2013; Klann and Dever, 2004). In addition, aberrant local translation is a feature of several neuropsychiatric and neurodegenerative disorders (Liu-Yesucevitz et al., 2011; Donlin‐Asp et al., 2017). Therefore, it is vital to our understanding of brain function that we clarify the mechanisms that govern local synaptic translation in health and disease.

Due to the technical challenges of studying local translation, there is no definitive answer yet regarding the precise composition of the synaptic translatome (transcripts in a cell or tissue, which may be translated at a given point in time) and how it is regulated (Rangaraju et al., 2017). The translatome differs from the proteome, since it represents the pool of mRNAs that are associated with translating ribosomes. Thus, changes in ribosome occupancy may not precisely reflect changes in the proteome. However, it has been shown on several instances that measuring ribosome occupancy allows for more confident estimations of protein expression than transcriptome analysis (RNA-Seq or microarrays) (Carlyle et al., 2018; Schwanhäusser et al., 2011; Vogel, 2011). To study local translation, several approaches have been employed, including microdissection of the neuropil layer within the hippocampal CA1 region from fresh tissue (Tushev et al., 2018), fluorescence activated synaptosome sorting (FASS) combined with RNA-Seq (Hafner et al., 2018), and the use of tripartite microfluidic systems that separate the soma, axon, and pre-synapse of cultured neurons into different compartments (Baleriola et al., 2014). Although useful to get an insight into the local translatome, these methods have several limitations. While microdissected tissues contain significant contaminations from glia and other non-neuronal cells, which require extensive bioinformatics analysis to eliminate (Tushev et al., 2018), culture systems fail to mimic the complexity of *in vivo* neuronal networks and are limited to the specific brain regions and development ages from which stable neuronal cultures can be obtained. Furthermore, recent approaches have employed Translating Ribosome Affinity Purification (TRAP), but despite the fact that they offer cell-type specificity, they require genetic manipulation to introduce an exogenous tag (Green Fluorescent Protein) on ribosomal proteins (Eacker et al., 2017; Ouwenga et al., 2017).

Here, we present the combination of a well-established method for synaptoneurosome (SN) preparation (adapted from Dunkley et al., 2008) with the ribosome profiling methodology (Ingolia et al., 2009, 2012). SN are biochemically isolated pre- and post-synaptic components that are obtained through gentle homogenisation of nervous tissue under isotonic conditions. These preparations have the advantage that they can be rapidly isolated from any brain region, from animals of any age, and have little contamination from non-synaptic components and non-neuronal cell types. Ribosome profiling is an unbiased RNA sequencing-based strategy to assess the transcriptional and translational, i.e. their association with translating ribosomes, state of cells. Therefore, our method allows the study of the synaptic translatome from whole brain tissue or brain regions of interest in an unbiased way.

## 2 Methods

### 2.1 Animals

C57Bl/6 J mice were bred and maintained within animal facilities of the University of Edinburgh. Animals were weaned at postnatal day 21 and thereafter housed in cages of up to 4 animals, in temperature (20–21 °C) and humidity (∼55%) controlled rooms. Animals were kept on a 12 h light/dark cycle and had access to food and water *ad libitum*. All experimental procedures were performed in accordance with UK Home Office regulations.

### 2.2 Preparation of synaptoneurosomes (SN)

Synaptoneurosomes were prepared from the forebrain (whole brain dissection-olfactory bulbs and cerebellum were removed) of 10-week-old C57Bl/6 J male mice.

**Fig. 1:**
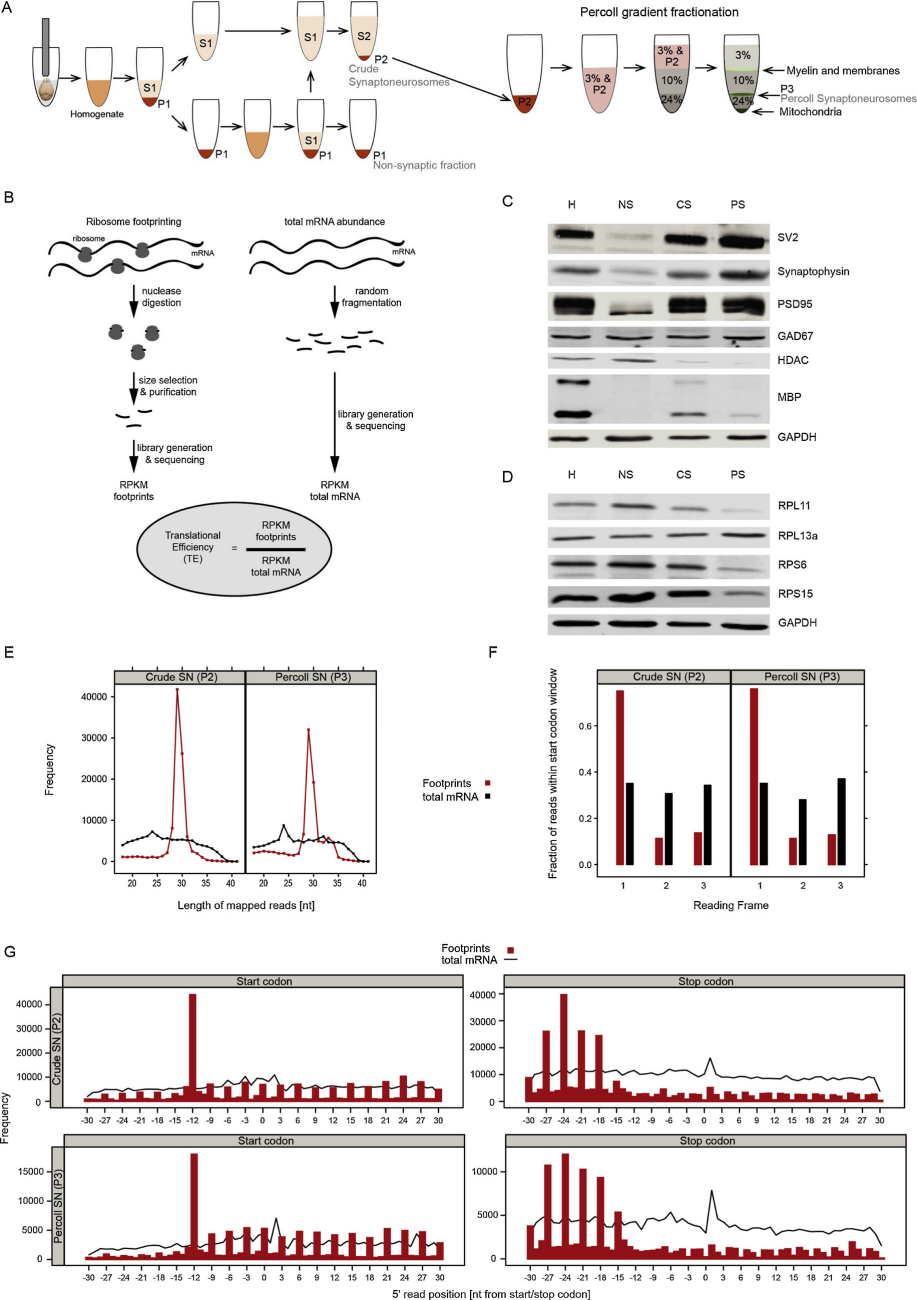
Schematic diagram of the preparation of SN (A) and the ribosome profiling workflow (B). A detailed description can be found in the Methods section. (C) Immunoblots of the indicated proteins in the different fractions obtained during the preparation of SN. Note the depletion of nuclear proteins (HDAC) and the enrichment in both excitatory and inhibitory synaptic proteins (synaptic vesicle glycoprotein 2A - SV2, synaptophysin, glutamic acid decarboxylase 67 – GAD67, and postsynaptic density protein 95 - PSD95) in crude and Percoll SN. PSD95 blots show two bands very close in size, representing α and ß isoforms of the protein, respectively (Chetkovich et al., 2002). In addition, Percoll SN show a major decrease in myelin components (MBP) of both major isoforms. GAPDH was used as a loading control. H: tissue homogenate, NS: non-synaptic fraction, CS: crude synaptoneurosomes, PS: Percoll synaptoneurosomes. (D) Immunoblots confirming the presence of ribosomal proteins (large ribosomal proteins 11 and 13a, small ribosomal proteins 6 and 15) in the SN fractions. GAPDH was used as a loading control. (E) Size distribution of the aligned sequencing reads from the indicated samples. Total mRNA reads show a random size distribution, whereas ribosomal footprints show a distinct peak between 28 and 30 nt. (F) Reading frame usage in the total mRNA and footprint samples, showing the preferential alignment within the first reading frame in the footprint samples, compared to the total mRNA samples which have been randomly fragmented. (G) The total number of read fragments aligning around the start and stop codons of the coding sequence of all genes. Footprints show a 3 nt periodicity, compared to total mRNA reads.

Crude SN and Percoll SN were prepared as described in (Dunkley et al., 2008), with minor modifications (Fig. 1A). Briefly, the freshly dissected forebrain was homogenized in ice–cold sucrose buffer (320 mM sucrose, 5 mM Tris-HCl pH 7.4, 1 mM EDTA) and centrifuged for 10 min, at 1000x *g*, 4 °C. The pellet (P1) was resuspended in sucrose buffer and centrifuged as before. The resulting pellet (P1) was kept as the “non-synaptic fraction” and the combined supernatant from both spins (S1) was further centrifuged for 10 min at 21,000x *g*, 4 °C, to pellet crude SN (P2).

To prepare Percoll SN, the crude pellet (P2) was resuspended in 3% Percoll (GE Healthcare, 17089101) in sucrose buffer and centrifuged through a discontinuous 10%–24% Percoll gradient, at 30,750 x *g* for 9 min at 4 °C, with minimum acceleration and no deceleration on a JA-25.50 fixed angle rotor in a Beckman Avanti JA-25 centrifuge. The material between layers 24% and 10% was collected, resuspended in Ionic Media (20 mM HEPES pH 7.4, 10 mM Glucose, 1.2 mM Na_2_HPO_4_, 1 mM MgCl_2_, 5 mM NaHCO_3_, 5 mM KCl, 140 mM NaCl), and centrifuged for 15 min at 21,000 x *g*, 4 °C. Cycloheximide (100 μg/ml) was added to all buffers to stall translating ribosomes and stabilise them in their position on the mRNA.

### 2.3 Western blotting

Protein was extracted from samples by addition of RIPA buffer [50 mM Tris-HCl pH 8.0, 150 mM NaCl, 1% NP-40, 0.5% sodium deoxycholate, 0.1% SDS, protease and phosphatase inhibitors (Roche, cOmplete and PhosSTOP mini tablets)] and homogenisation using a motorized pestle (Kimble, 749540-0000). Samples were incubated on ice for 15 min, with occasional vortexing, and centrifuged for 20 min at 16,000 x *g* at 4 °C to clear the lysate from cellular debris. The protein concentration of each sample was determined by Bradford assay (Bio-Rad, 5000112).

Samples were prepared in SDS sample buffer (50 mM Tris pH 6.8, 100 mM DTT, 2% SDS, 10% Glycerol, 0.1% bromophenol blue), heated for 5 min to 95 °C and resolved on polyacrylamide gels. Proteins were transferred to 0.2 μm nitrocellulose membranes (Bio-Rad, 1620112), blocked in 5% BSA in TBS-T (10 mM Tris pH 7.6, 150 mM NaCl, 0.1% Tween-20) for 1 h at room temperature (RT), incubated with primary antibodies in 1% BSA in TBS-T overnight at 4 °C, and with secondary antibodies in 1% BSA in TBS-T for 1 h at RT. Between incubations, membranes were washed extensively in TBS-T. Blots were imaged using an Odyssey Imaging System (Li-COR Biosciences) at a resolution of 169 μm.

Antibodies used for Western blotting are summarized in Table 1, Table 2.

**Table 1:** Details of primary antibodies used.

Target	Species	Supplier	Cat No	Dilution
GAD67	mouse	Millipore	MAB5406	1:1000
GAPDH	rabbit	Cell Signalling	2118	1:5000
HDAC3 7G6C5	mouse	GeneTex	GTX83173	1:1000
Myelin Basic Protein	mouse	abcam	ab62631	1:1000
PSD95	rabbit	Cell Signalling	3450	1:1000
Ribosomal Protein L11	rabbit	Cell Signalling	18163	1:1000
Ribosomal Protein L13a	rabbit	Cell Signalling	2765	1:500
Ribosomal Protein S6	mouse	Santa Cruz	sc-74459	1:5000
Ribosomal Protein S15	rabbit	abcam	ab157193	1:1000
SV2A	mouse	DSHB University of Iowa	AB_2315387	1:1000
Synaptophysin 1	rabbit	Synaptic Systems	101 002	1:1000

**Table 2:** Details of secondary antibodies used.

Description	Species	Supplier	Cat No	Dilution
IRDye® 680RD Donkey anti-Rabbit IgG (H + L)	Donkey	Li-COR Biosciences	926-68073	1:5000
IRDye® 800CW Donkey anti-Mouse IgG (H + L)	Donkey	Li-COR Biosciences	926-32212	1:5000

### 2.4 Ribosome profiling

Ribosome profiling was adapted from (Ingolia et al., 2009, 2012) for use with the TruSeq Ribo Profile (Mammalian) Kit (Illumina, RPMHMR12126) and the NEXTflex™ Small RNA Sequencing Kit v3 (Bioo Scientific, NOVA-5132-06). The TruSeq Ribo Profile protocol was followed until the end-repair step, after which the NEXTflex™ Small RNA Sequencing Kit v3 was used for generating sequencing libraries. Polysomes were extracted from SN preparations through homogenisation in TruSeq Polysome Buffer (Illumina). A fraction of the lysates was kept as an internal mRNA control (total mRNA), while the remaining fraction was digested with TruSeq Ribo Profile Nuclease (RNase I) (footprints) and purified through a MicroSpin S-400 column (GE Healthcare, 27514001) to enrich for small RNA fragments. From synaptoneurosomes prepared from one full forebrain (one animal), we extracted between 600 ng and 4 μg of RNA after this extraction step, depending on the sample type (footprints usually show lower yield than total RNA). All samples (footprints and total mRNA) went through a ribosomal RNA depletion protocol using the Ribo-Zero Gold (Human/Mouse/Rat) Kit (Illumina, MRZG12324). The total mRNA was heat-fragmented according the TruSeq Ribo Profile Kit, whereas the footprint samples were further purified on a 15% TBE-Urea polyacrylamide gel (ThermoFisher Scientific, EC68852BOX) to select for bands running between 28 and 30 nucleotides. The quality and concentration of samples was assessed by running an Agilent Small RNA chip Bioanalyzer assay (Agilent Technologies, 5067-1548), which allows to assess the size distribution and concentration of RNA samples sized below 200 nt using as little as 50 pg of RNA sample. Libraries were generated using the NEXTflex™ Small RNA Sequencing Kit v3, according to the manufacturer’s instructions. The NEXTflex™ Small RNA Sequencing Kit v3 is particularly suitable for these applications, as library generation is successful even at very low input levels (we have used as little as 3 ng of purified, rRNA depleted footprints as total input). Synaptoneurosomes can also be prepared from specific brain regions, in which case we would recommend pooling tissue from more than one animal to achieve comparable quantities of RNA. Bioinformatics analysis was performed as previously described (Amorim et al., 2018). Translational Efficiency (TE) was calculated as the ratio between reads per kilobase per million mapped reads (RPKM) of footprints and RPKM of total mRNA for each gene.

Note: *Since the submission of this manuscript, the two Illumina kits (RPMHMR12126, MRZG12324) have become obsolete*. *We recommend following the original published protocol (**Ingolia et al., 2012**), but scaling down volumes for the footprinting step (to 100 μl), replacing the sucrose cushion with MicroSpin S-400 purification (as summarized in the Illumina protocol), and using a commercial kit for the rRNA depletion (e.g. NEBNext® rRNA Depletion Kit, New England Biolabs, E6350).*

To confirm validity of our data set, we determined the overlap between the 1000 most abundant transcripts in our SN preparations and a published list of mRNAs identified in SN prepared from adult mouse hippocampi (You et al., 2015).

### 2.5 Gene ontology analysis

Gene Ontology (GO) analysis was performed using the online tool DAVID (Database for Annotation, Visualization and Integrated Discovery; version 6.8) (Huang et al., 2009). Filtered gene lists were submitted to DAVID and GO annotations gathered for Biological Function, and Cellular Component.

## 3 Results

### 3.1 Isolation of synaptically enriched fractions

We prepared SN from mouse forebrain using two different methods, which yield synaptically enriched fractions of different purity levels. Crude SN offer a quick and simple way of isolating synaptically enriched fractions from brain tissue. However, even though this method might be suitable for certain purposes, crude SN are contaminated with myelin, non-synaptic mitochondria, and other cellular debris. If a purer sample is required, crude SN can be centrifuged through discontinuous Percoll gradients to remove most contaminants (Fig. 1A).

As demonstrated by immunoblotting (Fig. 1C), our protocol reliably produced synaptic fractions depleted of nuclear components and enriched in both excitatory and inhibitory synaptic proteins. SN purified from PErcoll gradients were also depleted of further contaminating cellular components, such as myelin basic protein (MBP, two isoforms (Harauz and Libich, 2009)). Furthermore, we probed our samples with antibodies against ribosomal proteins to show that there are adequate levels of ribosomal proteins present in synaptic fractions (Fig. 1D).

### 3.2 Ribosome profiling in synaptoneurosomes

Having successfully isolated synaptic fractions from brain tissue, we proceeded with processing them for ribosome profiling (Fig. 1B). The crude and Percoll SN were lysed and separated into two fractions, in order for total mRNA (a proxy for transcription) and footprints (a proxy for translation), to be assessed simultaneously within each sample. For footprint analysis, the samples were digested with RNase I, to generate footprints of around 28–30 nucleotides (nt) in length. Total mRNA was heat-fragmented to yield sequence fragments similar in size to footprints. Both sets of samples were run through a ribosomal RNA removal kit, to eliminate highly abundant ribosomal RNA contaminants, and sequencing libraries were prepared from footprints and total mRNA fragments.

We succeeded in generating high quality ribosome profiling libraries from both crude and Percoll SN (Fig. 1E–G). Quality control graphs confirmed the restricted size distribution of footprints, as well as the random size of total mRNA fragments (Fig. 1E). Analysis of the usage of reading frames showed that the majority of footprints aligned with their corresponding main reading frame sequence (Fig. 1F, Frame 1), as would be expected for actively translating ribosomes. In addition, mapping of the footprints along each transcript showed that the 5′-ends of the footprints start at 12 nt upstream of start codons and decrease significantly in frequency at approximately 15 nt upstream of the stop codon, specific to the size and shape of initiating and terminating ribosomes, respectively. The footprints further exhibit a characteristic 3-nt periodicity (Fig. 1G), a consequence of translating in 3-nt codons. In contrast, the equal distribution of total mRNA fragments between the 3 main reading frames and their lack of 3-nt nucleotide periodicity highlights the random nature of the heat fragmentation process and the specificity of the footprint data (Fig. 1F–G).

### 3.3 Gene ontology analysis

Sequencing identified transcripts from over 10,000 protein coding genes. Therefore, to facilitate further analysis and to focus on the most abundant transcripts in synapses, we selected the 1000 most abundant (top 1000) genes corresponding to the transcripts in our total mRNA and translational efficiency (TE) datasets with the highest RPKM or TE values, respectively, for both the crude and Percoll protocols.

**Fig. 2:**
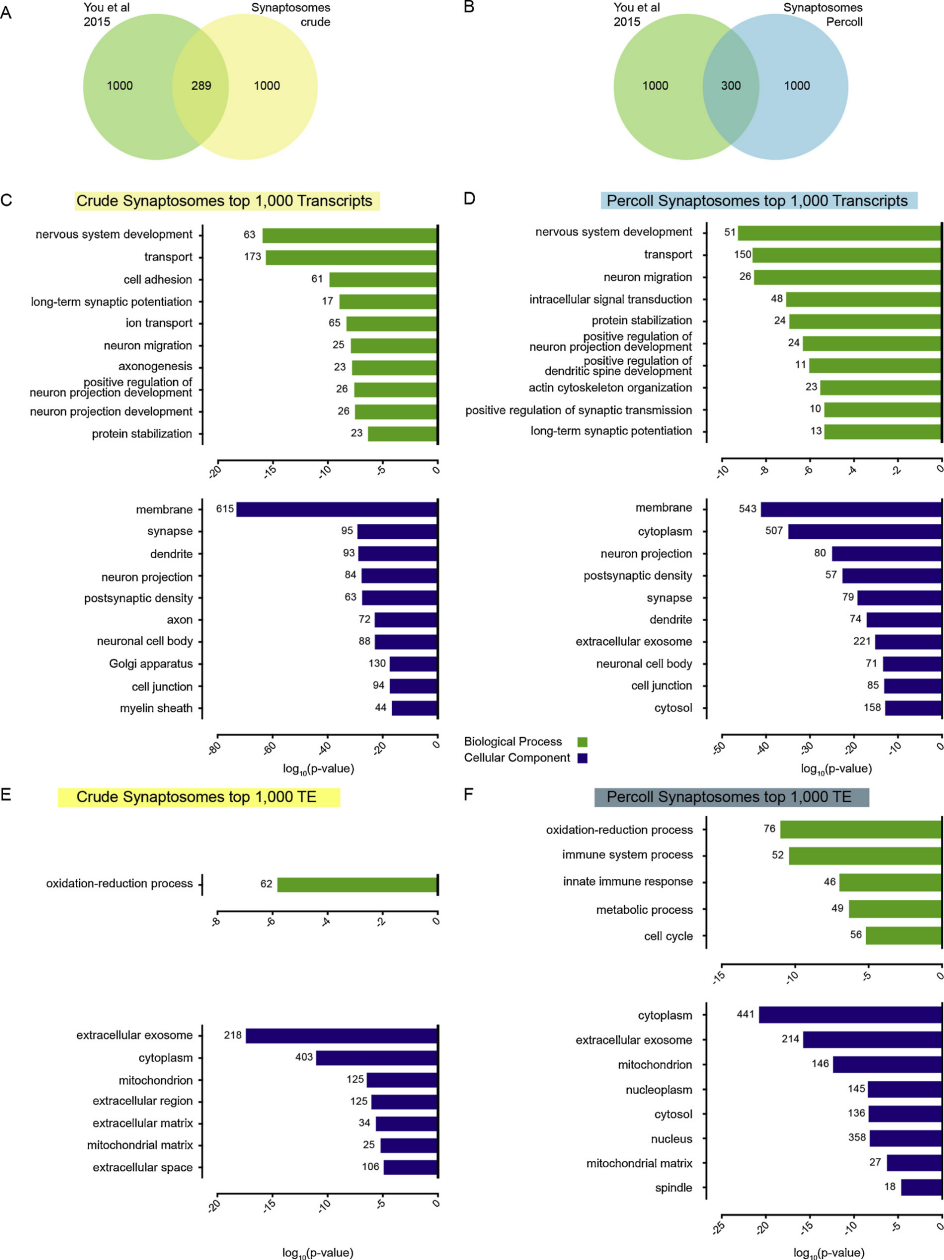
The overlap between the published data (You et al., 2015) and our crude and Percoll SN are 289 and 300 genes, respectively. (C–F) Most significant results from the DAVID GO analysis of the 1000 most abundant transcripts identified in the SN fractions (C–D), and the 1000 genes with the highest TE in the SN fractions (E–F). The numbers accompanying each bar on the graphs indicate the number of genes in the respective group.

First, we compared our data to a published dataset exploring the transcriptome of SN generated from mouse hippocampi (You et al., 2015) (Fig. 2A–B). Even though some regional heterogeneity would be expected when comparing SN isolated from hippocampus with those extracted from forebrain tissue, we found a ∼30% overlap for the top 1000 genes (total mRNA) for both the crude and Percoll SN. This shows that our data are comparable to data generated from similar experiments by other labs, and that there is a pool of highly abundant mRNAs enriched in synapses, which may support core synaptic functions across brain regions. Secondly, we performed Gene Ontology (GO) analysis of the top 1000 genes in our samples to define ontological groups of genes enriched in our SN preparations (Fig. 2C–F). Overall, performing GO analysis using DAVID revealed a highly significant enrichment (p < 0.0001) of Biological Process and Cellular Component GO terms related to synaptic function in our gene lists (Fig. 2C–F). Total mRNA samples showed highly significant enrichments (p < 0.0001) in Cellular Components such as membrane, synapse, post-synaptic density and dendrites, emphasising the synaptic nature of the samples (Fig. 2C and D). Interestingly, TE datasets were particularly enriched (p < 0.0001) in transcripts coding for mitochondrial, extracellular matrix and extracellular exosome proteins (Fig. 2E and F). Biological Processes GO terms followed the same trends, with TE data showing enrichment for metabolic and oxidation-reduction processes (Fig. 2E and F), whereas the total mRNA data present an enrichment (p < 0.0001) in a broader range of important synaptic processes, including nervous system development, ion transport, neuron migration and long-term synaptic potentiation (Fig. 2C and D).

Taken together, these results show that our method reliably identifies transcripts localised to the synapse, as well as the subset of these mRNAs that are associated to ribosomes and are likely to be locally translated.

## 4 Discussion

Here we present a new methodology based on two previously established protocols, allowing us to study simultaneously the synaptic translatome/transcriptome of neurons. We further demonstrate that our method yields high quality RNA sequencing data displaying an enrichment in genes related to synaptic function.

Studying synaptic translation in an unbiased way poses a great challenge, due to the technical difficulty of isolating pure dendritic or axonal fractions containing mRNA (Rangaraju et al., 2017). Ribosome profiling has been previously used to study translation in the rodent brain (Amorim et al., 2018; Cho et al., 2015) and provides an unbiased, genome-wide assessment of both the total transcriptome and translatome. Studying the translatome is important because translation contributes significantly to regulating protein levels, especially at synapses (Vogel, 2011). By purifying synaptic compartments from live brain tissue, using a well-established method, we were able to generate ribosome profiling libraries, which allow for gaining a significant insight into the transcriptome and translatome of SN.

By performing GO analysis of the top synaptic transcribed and translated mRNAs, we showed an enrichment in synaptically relevant transcripts in both the total mRNAs and the ribosome associated fractions, as well as an overlap with comparable published data (Fig. 2A and B). GO analysis of our transcriptome targets (Fig. 2C and D) showed significant enrichment in Cellular Component terms relating to the synapse (post synaptic density, dendrite, synapse, axon, etc) and Biological Process terms such as long-term synaptic potentiation and ion transport. Interestingly, GO analysis of the most abundant genes in the ribosome associated fraction (TE dataset) showed an enrichment in energy metabolism- and mitochondria-related terms (Fig. 2E and F). This is in agreement with the high abundance of mitochondria at the synapse and the elevated energetic demand required for the maintenance of synaptic functions (Rangaraju et al., 2019). The contrast between total mRNA and TE data highlights the fundamental difference between the transcripts present at synaptic fractions and their level of translation, stressing the importance of assessing both the transcriptome and translatome of a given sample.

The choice between using crude or Percoll SN depends on the requirements of each particular experiment. Both preparations are enriched in synaptic components and in ribosomal proteins (Fig. 1C and D) and RNA. Total mRNA and footprint libraries can be prepared from either type of sample, and GO analysis shows similar enrichments in relevant synaptic-related terms (Fig. 2). Percoll SN have the advantage of being less contaminated with myelin and extra-synaptic mitochondria (Fig. 1C, Fig. 2C), but require longer preparation times and the use of freshly dissected tissue. Percoll SN yield lower amounts of mRNA and footprints than crude SN, which may require pooling of samples from different animals for small brain regions. Ultimately, the researcher needs to take into consideration the balance between the purity of samples and technical feasibility of the experiment.

Combining SN isolation with translational profiling has the potential of answering important questions within the field of localised neuronal translation in an unbiased fashion. SN can be isolated from whole brain, prepared from specific brain regions or sorted using flow cytometry to yield specific populations (Hafner et al., 2018; Biesemann et al., 2014). In addition, SN are commonly used as functional *in vitro* models of synaptic activity, which can be assessed at baseline or can easily be stimulated or treated with a variety of agents. The combination of this powerful tool with ribosome profiling allows researchers to precisely analyse the local translatome at synapses and to study translational regulation in response to neuronal activity or in models of neurological disorders.

## 5 Conclusion

The combination of the unbiased method of ribosome profiling, to study transcripts that are being actively translated, with well-established protocols of isolating functional synaptic fractions provides a powerful tool to study localised translation at the synapse.

## Funding

This work was supported by grants to C.G.G.: Sir Henry Dale Fellowship from the Wellcome Trust and Royal Society (107687/Z/15/Z), a NARSAD Young Investigator grant from the Brain & Behavior Research Foundation (24968) and a grant from the Simons Initiative for the Developing Brain.

## Declaration of Competing Interest

The authors declare no competing financial interests.
